# The complete chloroplast genome sequence of *Potentilla glabra* Lodd

**DOI:** 10.1080/23802359.2021.1934141

**Published:** 2021-06-07

**Authors:** Song Yu, WenJuan Kang, Fang Yang, FuXin Li

**Affiliations:** a Medical College, Qinghai University, Xining, PR China; b State Key Laboratory of Plateau Ecology and Agriculture, Qinghai University, Xining, PR China

**Keywords:** *Potentilla glabra* Lodd, chloroplast genome, phylogenetic analysis

## Abstract

*Potentilla glabra* Lodd is an important traditional Chinese medicine, which is widely distributed in many areas of China. It has a variety of pharmacological activities and has been drunk as a tea for a long time. Therefore, it has great economic, research, and social value. However, no plastid genome has been reported to date. Here, we report species of this genus complete chloroplast genome of *Potentilla glabra* Lodd. The chloroplast genome of *Potentilla glabra* Lodd is found to be 152,900 bp in length with 37.24% GC contents. The cp genome sequences contained 130 genes, including 84 mRNA genes, 37 tRNA, eight rRNA genes, and one pseudogenes, respectively. A phylogenetic tree reconstructed by 42 chloroplast genomes reveals that *Potentilla glabra* Lodd is most related with *Potentilla parvifolia*. The complete chloroplast genome of these plants will be benefit for studies on the general characteristics and evolution of the Potentilla family genome.

*Potentilla glabra* Lodd (*P. glabra*) belongs to the diverse *Potentilla* genus of Rosaceae, which is mostly found in mountains, valleys, and forests at higher altitudes, mainly distributed in Northeast, North China, Loess Plateau, and Southwest China. North Korea, the Soviet Union, and Mongolia are also distributed (Editorial Committee of flora of China, Chinese Academy of Sciences 1985). *P. glabra* is widely used for high blood fat, high blood pressure, diabetes, insomnia, upset, and softening blood vessels, etc. It can be used as a tea for long-term drinking (Bai et al. 2012; Han et al. 2016). According to the study of pharmacological activity, it has antiviral, antioxidant, antibacterial, and immunomodulatory activities (Hu et al. 2013; Wang et al. 2013). Therefore, it has high development and utilization value. A complete chloroplast genome data will contribute to the study and development of this plant. In this study, we assembled and characterized the complete chloroplast genome sequence of *P. glabra* using the genome skimming sequence data in order to provide a theoretical basis for the study and conservation of *P. glabra.*

In this study, we reported the completed chloroplast genomes of *P. glabra*. The fresh leaves were collected from a plant in Changning Town, Datong County, Xining City, Qinghai Province, China (N36°55′59.17″ and E101°41′45.95″). The specimen was deposited at the herbarium of pharmacy department of Qinghai University, the specimen accession number is 6312120082000LY. Genomic DNA was extracted following the modified CTBA method (Doyle 1987). The complete chloroplast genome was sequenced by Illumina HiSeq2500 platform (Illumina Inc., San Diego, CA), assembled with SPAdes version 3.10.1 (Bankevich et al. 2012) and annotated with CpGAVAS (Liu et al. 2012). Phylogenies trees were generated by maximum-likelihood (ML) analysis. The sequences were multiple sequence alignment using MAFFT, choose the GTR and hill-climbing model, with the ML inference with 100 bootstrap replicates by RaxML-HPC2 on TG version 7.2.8 on the Cipres web server (Stamatakis et al. 2008). A General Time Reversible was selected as the best substitution models for the ML.

The complete chloroplast genome sequence of *P. glabra* (MW092109) was 152,900 bp in length. The typical quadripartite structure consists of a pair of IRs of 25,311 bp, an LSC region of 84,148 bp, and SSC region of 18,130 bp, respectively. The overall GC content of *P. glabra* chloroplast genome is 37.24% and the corresponding values in LSC, SSC, and IR regions are 35.16, 31.09, and 42.90%, respectively. The genome was predicted to contain 130 genes, including 84 protein-coding genes, 37 tRNA genes, eight rRNA genes, and one pseudogenes.

A total of 220 SSRs microsatellites were identified, and there were 146, 10, 62, and 2 mono-, di-, tri-, and tetra-nucleotides repeats, respectively. Mononucleotide SSRs were the richest (66.36%). All the protein-coding genes presented a total of 26,222 codons, leucine (2761 codons, approximately 10.53% of the total) being the most abundant amino acid. Isoleucine (2240 codons, approximately 8.54%).

ML analysis showed that *P. glabra* formed a small branch with *Potentilla parvifolia*, belonging to *Potentilla* genus of Rosaceae. Among them, *P. glabra* is most related to *Potentilla parvifolia* in Rosaceae, with bootstrap support values of 100% (Figure 1). The complete chloroplast date of *P. glabra* can provide reference for taxonomic study of this genus, and it also provides useful information for the conservation and study of this multi-purpose natural resource.

**Figure 1.**
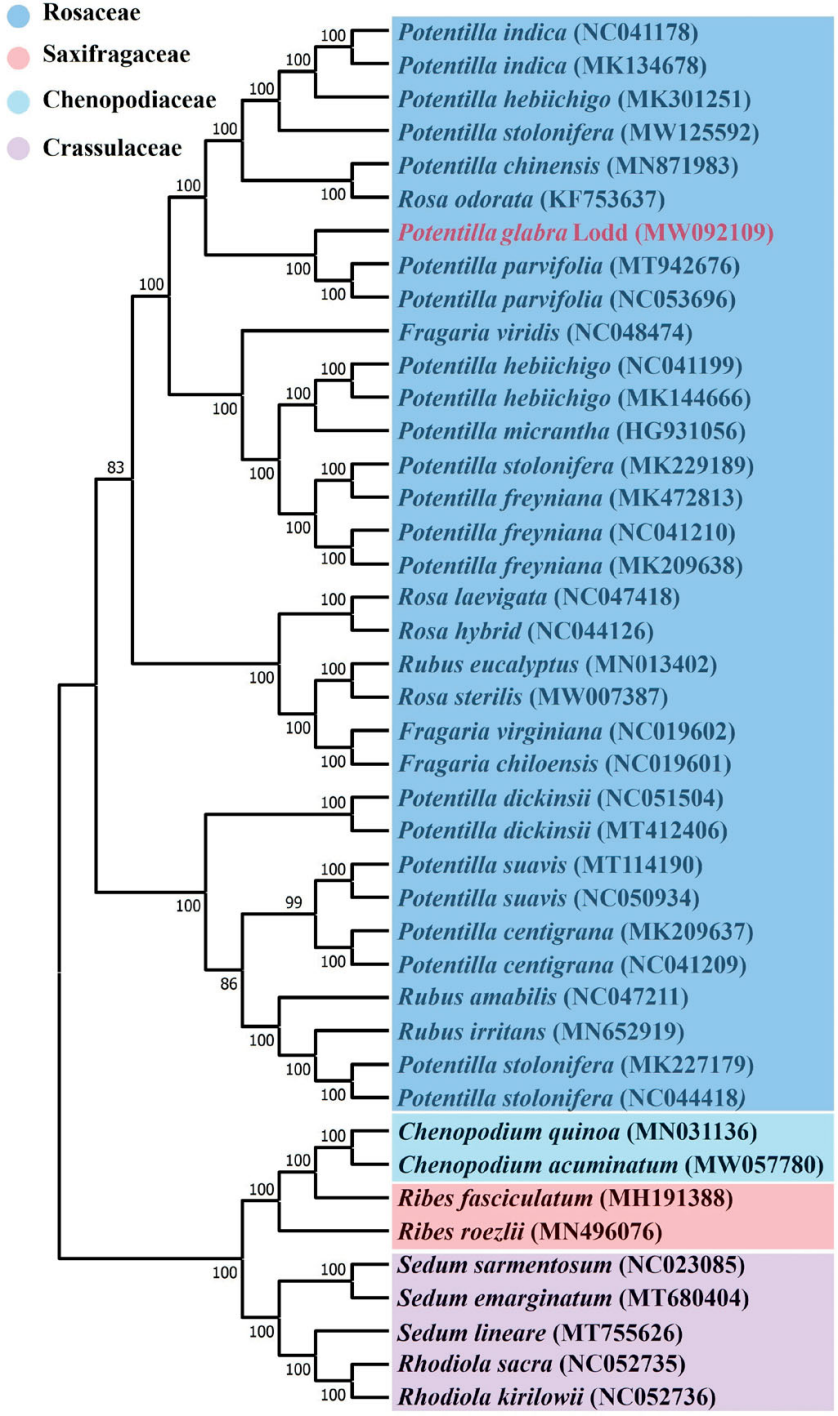
Maximum-likelihood phylogenomic tree based on 42 complete chloroplast genome sequences. The *Potentilla glabra* Lodd is marked in red and bootstrap values are listed for each branch.

## Data Availability

The data that support the findings of this study are openly available in NCBI GenBank at https://www.ncbi.nlm.nih.gov/nuccore/MW092109.1/, reference number MW092109.
